# Characterizing the insecticide resistance of *Anopheles gambiae* in Mali

**DOI:** 10.1186/s12936-015-0847-4

**Published:** 2015-08-22

**Authors:** Moussa B. M. Cisse, Chitan Keita, Abdourhamane Dicko, Dereje Dengela, Jane Coleman, Bradford Lucas, Jules Mihigo, Aboubacar Sadou, Allison Belemvire, Kristen George, Christen Fornadel, Raymond Beach

**Affiliations:** PMI Africa Indoor Residual Spraying Project, Abt Associates, Mali, Cite du Niger. BP: 34, Bamako, Mali; National Malaria Control Programme, Badalabougou, Rue 108 Porte 106, Bamako, Mali; PMI Africa Indoor Residual Spraying Project, Abt Associates, 4550 Montgomery Ave, Suite 800 North, Bethesda, MD 20814 USA; President’s Malaria Initiative USAID, ACI2000, Rue 243, Porte 297-BP 34, Bamako, Mali; President’s Malaria Initiative USAID, 1300 Pennsylvania Avenue NW, Washington DC, USA; Division of Parasitic Diseases and Malaria, Center for Global Health, US Centers for Disease Control and Prevention, Atlanta, GA 30333 USA

**Keywords:** *Anopheles gambiae*, Insecticide-based malaria vector control, Vector-insecticide resistance, Resistance monitoring, Mali

## Abstract

**Background:**

The impact of indoor residual spraying (IRS) and long-lasting insecticide nets (LLINs), key components of the national malaria control strategy of Mali, is threatened by vector insecticide resistance. The objective of this study was to assess the level of insecticide resistance in *Anopheles gambiae* sensu lato populations from Mali against four classes of insecticide recommended for IRS: organochlorines (OCs), pyrethroids (PYs), carbamates (CAs) and organophosphates (OPs). Characterization of resistance was done in 13 sites across southern Mali and assessed presence and distribution of physiological mechanisms that included target-site modifications: knockdown resistance (*kdr)* and altered acetycholinesterase (*AChE*), and/or metabolic mechanisms: elevated esterases, glutathione S-transferases (GSTs), and monooxygenases.

**Methods:**

The World Health Organization (WHO) tube test was used to determine phenotypic resistance of *An.gambiae s.l.* to: dichlorodiphenyltrichloroethane (DDT) (OC), deltamethrin (PY), lambda-cyhalothrin (PY), bendiocarb (CA), and fenitrothion (OP). Identification of sibling species and presence of the *ace*-*1*^*R*^ and Leu-Phe *kdr*, resistance-associated mutations, were determined using polymerase chain reaction (PCR) technology. Biochemical assays were conducted to detect increased activity of GSTs, oxidases and esterases.

**Results:**

Populations tested showed high levels of resistance to DDT in all 13 sites, as well as increased resistance to deltamethrin and lambda-cyhalothrin in 12 out of 13 sites. Resistance to fenitrothion and bendiocarb was detected in 1 and 4 out of 13 sites, respectively. *Anopheles coluzzii*, *An. gambiae* sensu stricto and *Anopheles arabiensis* were identified with high allelic frequencies of *kdr* in all sites where each of the species were found (13, 12 and 10 sites, respectively). Relatively low allelic frequencies of *ace*-*1*^*R*^ were detected in four sites where this assessment was conducted. Evidence of elevated insecticide metabolism, based on oxidase, GSTs and esterase detoxification, was also documented.

**Conclusion:**

Multiple insecticide-resistance mechanisms have evolved in *An. coluzzii*, *An. gambiae s.s.* and *An.arabiensis* in Mali. These include at least two target site modifications: *kdr*, and *ace*-*1*^*R*^, as well as elevated metabolic detoxification systems (monooxygenases and esterases). The selection pressure for resistance could have risen from the use of these insecticides in agriculture, as well as in public health. Resistance management strategies, based on routine resistance monitoring to inform insecticide-based malaria vector control in Mali, are recommended.

## Background

Recent evidences from survey data indicated that the scale-up of malaria interventions across sub-Saharan Africa has contributed to a reduction in under-five mortality [1]. The contribution of vector control measures, long-lasting insecticide nets (LLINs) and indoor residual spraying (IRS), to this effort cannot be overestimated, and should continue (assuming adequate resources) as long as vector populations remain susceptible to ‘public health’ insecticides [2, 3]. The susceptibility ‘condition’ for ongoing impact is by no means assured because of the small number of public health insecticide classes available. These include four classes for IRS (organochlorines (OCs), organophosphates (OPs), carbamates (CAs), and pyrethroids (PYs), and of even greater concern, only one, PYs, for use on nets.

The development and spread of insecticide resistance in the populations of *Anopheles gambiae* sensu lato (*s.l.*), a major vector of malaria in Africa, presents a serious threat to the progress made in malaria control. Extensive use of insecticides in agriculture and the scale-up of insecticide-based malaria vector control during the past decade appear to have played a pivotal role in the emergence and rapid spread of insecticide resistance on the continent [4, 5]. Resistance, especially to PY insecticides and dichlorodiphenyltrichloroethane (DDT), in *An. gambiae s.l*., occurs across Africa [2, 6–8]. More recently, resistance to CA insecticides (bendiocarb and propoxur) and OPs (fenitrothion and malathion) has also been reported [2, 10, 11]. While few studies have assessed the public health impact of insecticide resistance, there is evidence of malaria vector control failure associated with pyrethroid resistance, [12, 13]. This threat may be more common than assumed since a recent systematic review and meta-analysis on the impact of pyrethroid resistance on the efficacy of LLINs points out that the heterogeneity of the studies masks relationships between resistance and control failure [14].

*Anopheles gambiae s.l.* populations show considerable heterogeneity in Mali. *Anopheles coluzzii*, *Anopheles arabiensis,* and *Anopheles gambiae* sensu stricto *(s.s.)* are present. Furthermore, there are at least two chromosomal forms of *An. gambiaes.s*.: Savanna and Bamako and a third one called Mopti that corresponds to *An. coluzzi* [15]. As early as 1987, the *kdr* allele was detected in the Savanna population from Bamako, and has increased in frequency over the years [16]. A more recent study on the spread of the *kdr* allele indicated a significant increase in frequency in the Savanna population and noted extension of the *kdr* allele to the Bamako chromosomal form for the first time [17].

The National Malaria Control Programme (NMCP) of Mali scaled up distribution of LLINs beginning in 2004, and is working towards universal coverage. Subsequently, IRS, also using PY class insecticides, was implemented in two districts: Bla and Koulikoro from 2008 to 2010, with a third district, Baraoueli, added in 2011, when CA insecticides were substituted for PY insecticides due to evidence of resistance in local vector populations [1]. Rotation to CAs (2011) was followed by another change in insecticide class, rotation to OPs in 2014 because of the short residual life of bendiocarb on muds walls. The increases in LLIN and IRS coverage in Mali, coupled with pesticide use in agriculture, have likely put selection pressure on malaria-transmitting mosquitoes, leading to an unfortunate emergence and spread of insecticide resistance [13].

The first step in managing resistance is to monitor its spread. Consequently, PMI has supported insecticide-resistance monitoring in Mali since 2007 and has documented the spread of resistance to DDT, PYs, and, most recently, CAs in *An. gambiae s.l.* in focal areas. Data from this effort form the basis of this report, which presents the current insecticide susceptibility/resistance status of *An. gambiaes.l.* populations at 13 sites across the central, south, and southwestern parts of the country. Additionally, we report on the presence and frequency of *kdr* and *ace*-*1*^*R*^ resistance mutations in *An. gambiae* sibling species, and assess the level and distribution of detoxifying enzymes, a second resistance strategy used by anopheline vectors. These results will help to mitigate the threat of resistance by informing a plan for resistance management and effective vector control interventions going forward.

## Methods

### Study area and duration

**Fig. 1:**
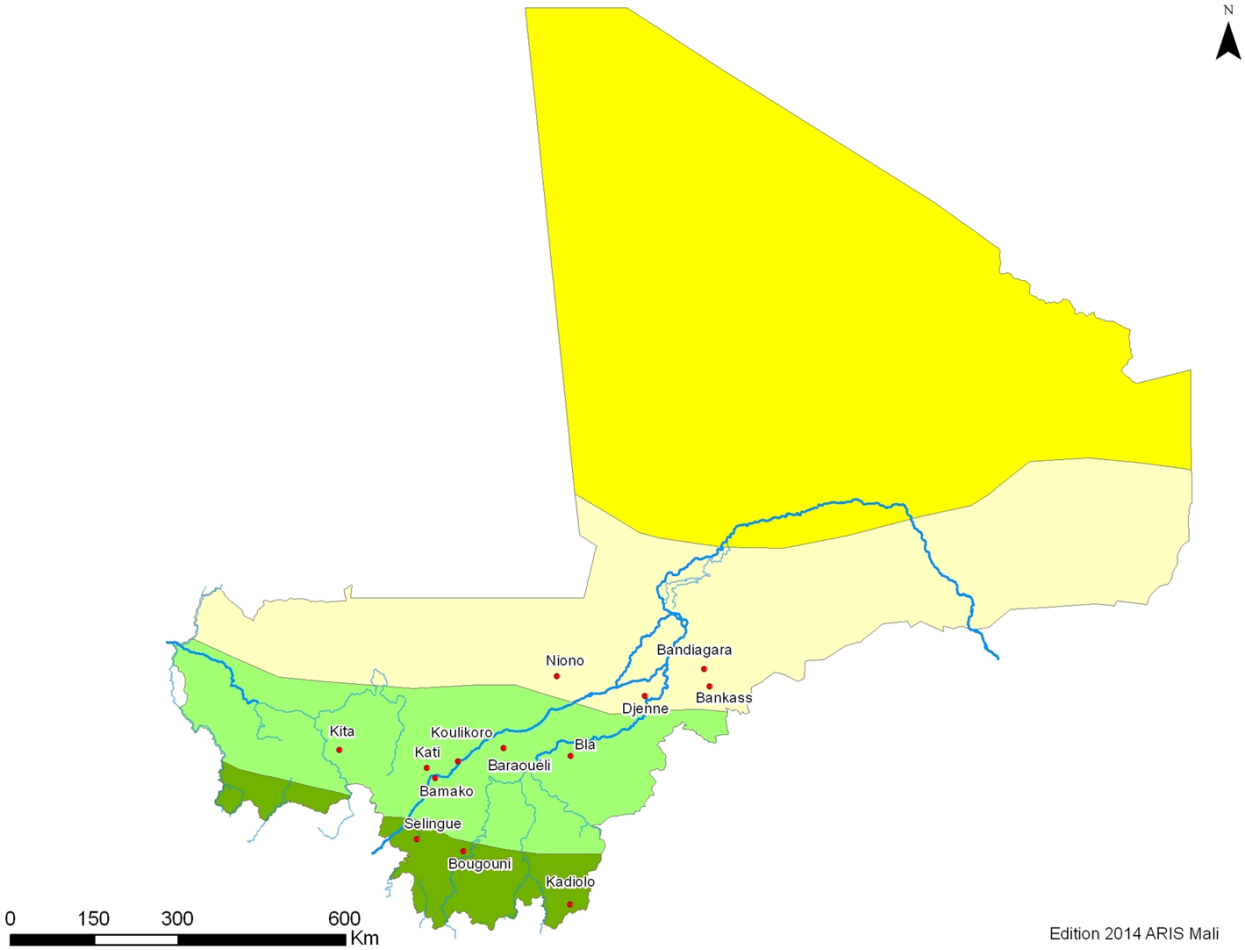
Eco climate map of Mali with insecticide surveillance sites

Altitudinal variation in our study area ranges from 200 to 350 m. The ‘rainy’ (peak malaria transmission) season (June–September) varies in length according to latitude, and alternates with a ‘dry’ season (October–May), that can include some rainfall. In 2012, insecticide resistance (bioassay) testing was done using *An. gambiae s.l.,* collected as larvae, between November and December. Collections were made at 13 sites (Table 1; Fig. 1) that had been selected based on factors such as: insecticide use for malaria control (IRS and LLINs) and agricultural crop protection, large-scale irrigation (supporting high vector density), and variation between ecological zones. The location and relevant eco-climatic zones for each site are shown in Fig. 1.

**Table 1:** Insecticide-resistance monitoring sites and their relevant characteristics

Region	District	Village	Factors considered in selection	Eco-epidemiological zone
Kayes	Kita	Fourgna Berda/Banfara	Agricultural insecticide use	Northern Sudanese short transmission season
Koulikoro	Koulikoro	Koulikoro	IRS	
	Kati	Kati	LLIN distribution/use Black fly control Irrigation—high	Northern Sudanese short transmission season
Segou	Niono	Sokourani/Toumakoro	Irrigation	Sahelian Flooded six-month seasonal transmission
	Bla	Tia, Touna	IRS	Northern Sudanese short transmission season
	Baraoueli	Bouadie/Tigui	IRS	
Sikasso	Bougouni	Massabla/Dalabani	Agricultural insecticide use	South Sudanese six-month seasonal transmission bi- or multi-modal
	Silengue	Binko	Irrigation	South Sudanese Flooded six-month seasonal transmission
	Kadiolo	Kadiolo	Agricultural insecticide use	South Sudanese six-month seasonal transmission
Mopti	Badiangara	Badiangara	Traditional agriculture (limited use of herbicides only)	Sahelian short seasonal transmission
	Bankass	Bankass		
	Djenne	Gomitogo/Djenne central/Wono/Edugu Were		Sahelian flooded six-month seasonal transmission
District of Bamako	Commune IV	Djicoroni Para	LLIN distribution/use	Northern Sudanese Suburban short transmission season

### Larval collection

Mosquitoes used in this assessment were field collected as larvae or pupae. Sampling was guided by the availability and the accessibility of larvae in ‘typical’ breeding sites of *An. gambiae s.l*., such as temporary pools of standing water, edges of slowly flowing rivers, and irrigation canals. While some collections were reared and tested in the field, others were brought back to the laboratory and reared to adulthood in an insectary, and then tested.

### Characterizing phenotypic resistance

The World Health Organization (WHO) standard tube bioassay test was used to characterize insecticide resistance. The test, performed according to WHO protocol [18], involves exposure of three- to five-day-old, non-blood-fed, female adults to a diagnostic dosage of insecticide: DDT (4 %), deltamethrin (0.05 %), lambda-cyhalothrin (0.05 %), bendiocarb (0.1 %) and fenitrothion (1 %) that kills ‘susceptible’ females, but allows ‘resistant’ individuals to survive. During the test, 94 to105 mosquitoes were exposed to the insecticide for 1 h. The field susceptibility data was collected in 2012, before the 2013 WHO test procedures that recommend vector exposure to fenitrothion (1 %) for 2 h was released in accordance with 1998 protocol. The exposure chambers (tubes) were held vertically, and each test included controls, where mosquitoes from the same population were ‘treated’ the same way, but without exposure to insecticide.

Deltamethrin and lambda-cyhalothrin testing was limited to only 4/13 and 9/13 sites, respectively, due to lack of enough impregnated papers to do testing at all sites. After 1 h of exposure, test mosquitoes were transferred to holding tubes without insecticide, and held for 24 h, when, mortality was recorded by visual inspection. The number of test females ‘alive’ was defined as observed percent of surviving test females after the 24-h holding period. Table 2 presents the interpretation of the test result in terms of resistance [19].

**Table 2:** Interpretation of WHO tube test results

Mean Mortality (%)	Mean survival	Resistance^a^	Susceptibility^a^
98–100	<2 %	No evidence of resistance (S)^b^	Population fully susceptible
90–97	>2 % but <10 %	Possible resistance (PS)^b^ (confirmation needed)	
<90	>10 %	Confirmed (R)^b^ assumed to be equal to % survival	

^a^Susceptibility and resistance considered as opposite interpretations of mortality

^b^Used in Table 3

### Characterizing physiological aspects of resistance

Following morphological identification [20] and phenotypic characterization, resistance in *An.gambiae s.l.* study populations was further characterized using biochemical and molecular methods [21–25]. Currently based on molecular and bionomical evidence, the *An. gambiae* molecular “M form” is named *An. coluzzii,* while the “S form” retains the nominotypical name *An. gambiae* [26]. All *An. gambiae s.l.* were identified to species by using PCR as described by Scott et al. [22]. *Anopheles coluzzii* and *An. gambiaes.s.* were then identified by PCR according to the protocol of Favia et al. [23].

Mixed-function oxidase (MFO), non-specific esterase (NSE) and glutathione S-transferase (GST) activity was assayed by spectrophotometry in individual two- to five-day-old adult females (not previously exposed to insecticide), according to the method described by Hemingway [21]. Tests were conducted on *An. gambiaes.l.* reared in the insectary, following collection at nine sites: Koulikoro, Kati, Bla, Niono, Kita, Silengue, Bougouni, Kadiolo, and Bandiagara. A total of 100 mosquitoes were processed for each assay per site except in Selingue where only 35 mosquitoes were processed for each of the assay.

All *An. coluzzii*, and *An. gambiae s.s.* were tested for the Leu-Phe *kdr* mutation according to the protocol of Martinez-Torres et al. [24]. The *ace*-*1*^*R*^ mutation was diagnosed by PCR- RFLP as described by Weill et al. [25].

### Data analysis and mapping

Biochemical assay data (enzymatic activity per mg protein) were compared between a susceptible, reference strain (Kisumu) and vectors from selected sites (9 of 13) by a Man Whitney non-parametric test. The association between the use of IRS at a study site and phenotypic resistance (WHO test result) was assessed using Poisson regression. The frequency of resistant alleles (*kdr* and *ace*-*1*^*R*^) between study sites was also compared using Poisson regression.

## Results

### Bioassay results by site

Based on WHO tube test results, resistance to DDT was observed in all 13 sites where *An. gambiaes.l.* populations were tested (Table 3). Relatively high levels of resistance (53–89 % resistance) and moderate levels of resistance (24–25 % resistance) to DDT were observed at 11 and two out of the 13 sites, respectively. Similarly, there was evidence for comparable resistance levels to lambda-cyhalothrin or deltamethrin (PY class insecticides), at 12 of the 13 surveillance sites. In contrast to the PY resistance observed, there is a possible resistance to deltamethrin in Bougouni, a result that requires additional testing prior to interpretation (Table 3).

**Table 3:** WHO insecticide susceptibility test results by district. *Anopheles gambaie s.l.* exposed to diagnostic concentrations of five insecticides representing the four classes of public health insecticides approved by WHO

District	Organochlorine		Pyrethroid				Carbamate		Organophosphate	
	DDT 4 %		Deltamethrin 0.05 %		Lambda-cyhalothrin 0.05 %		Bendiocarb 0.01 %		Fenitrothion 1 %	
	% Mortality (N)	Status	% Mortality (N)	Status	% Mortality (N)	Status	% Mortality (N)	Status	% Mortality (N)	Status
Kita	75 (101)	R			74 (100)	R	66 (96)	R	99 (100)	S
Koulikoro	11 (95)	R			13 (98)	R	98 (96)	S	99 (99)	S
Kati	17 (96)	R			12 (104)	R	100 (104)	S	100 (94)	S
Niono	44 (104)	R			33 (101)	R	100 (103)	S	93 (95)	PR
Bla	48 (99)	R			58 (102)	R	88 (102)	R	98 (119)	S
Baraoueli	13 (93)	R			33 (100)	R	98 (90)	S	97 (99)	PR
Bougouni	28 (103)	R	91 (103)	PR			85 (102)	R	97 (104)	PR
Silengue	23 (103)	R			50 (103)	R	100 (101)	S	100 (103)	S
Kadiolo	12 (105)	R			37 (101)	R	78 (103)	R	84 (100)	R
Badiangara	76 (105)	R	79 (106)	R			100 (104)	S	100 (105)	S
Bankass	47 (106)	R	32 (103)	R			100 (104)	S	100 (105)	S
Djenne	42 (98)	R	16 (105)	R			100 (103)	S	100 (101)	S
Bamako CIV	18 (98)	R			7 (104)	R	100 (104)	S	99 (103)	S

Sample size is in parenthesis (N)

*R* resistance, *PR* possible resistance, *S* susceptible

Bioassay results (% mortality <90 %) for *An. gambiae s.l.* from four local populations, Bla, Bougouni, Kita, and Kadiolo, indicated resistance to bendiocarb (Table 3). In contrast to the DDT and PY results, however, most of the *An. gambiae s.l*. populations tested (9 of 13) were fully susceptible to bendiocarb.

There was one *An. gambiae s.l.* fenitrothion-resistant population in Kadiolo and three populations with suspected resistance. The remaining nine populations were susceptible to fenitrothrion (Table 3).

Poisson regression was performed with mosquito mortality as the dependent variable and spray status as the covariate. Results from Poisson regression analysis indicated DDT susceptibility (% mortality) was significantly less in IRS areas as compared to areas with no IRS (P < 0.001). On the contrary, mosquito mortality when tested against bendiocarb (P = 0.769), fenitrothion (P = 0.904), and lambda-cyhalothrin (P = 0.7751) was not significantly associated with spray status.

### Levels of enzymes associated with resistance by site

Vector populations from Kati and Kadiolo had elevated α- and β-esterase levels, respectively one- and twofold higher when compared to females from a susceptible reference strain Kisumu (p < 0.05) (Table 4). Female *An. gambiae s.l*. from Kadiolo and Bandiagara showed one-fold higher oxidase levels (p < 0.05) and GST activity was elevated (p < 0.05) at eight sites: Koulikoro and Niono two-fold higher, Kati, Bla, Kita, and Selingue one-fold higher, Kadiolo three-fold higher, and Bandiagara four-fold higher (Table 4).

**Table 4:** Mean level of detoxifying enzyme activity in *Anopheles gambiae* *s.l.* mosquitoes collected from nine sites in Mali 2012

Strain	Mean activity			
	Alpha esterase	Beta esterase	Oxydases	GST
	µmol a-naph,/min/mg protein	µmol a-naph,/min/mg protein	Nmol P450/mg protein	Nmol GSH conj/min/mg protein
Kisumu	0.052	0.056	0.083	0.123
Koulikoro	0.061	0.059	0.071	0.251*
Kati	0.085*	0.099*	0.051	0.232*
Bla	0.035	0.045	0.053	0.236*
Niono	0.040	0.050	0.076	0.311*
Kita	0.026	0.037	0.071	0.176*
Selingue	0.038	0.061	0.064	0.221*
Bougouni	0.047	0.043	0.068	0.110
Kadiolo	0.117*	0.106*	0.162*	0.466*
Badiangara	0.034	0.039	0.155*	0.492*

Kisumu result expresses enzyme activity in susceptible reference strain

* p < 0.05: Enzyme level significant high compare to Kisumu strain

### Resistance gene (*kdr* and *ace*-*1*^*R*^) frequencies by site

A total of 1287 mosquitoes (855 *An. coluzzii,* 282 *An. gambiae s.s*., and 150 *An. arabiensis*) were genotyped for presence of the Leu–Phe (1014F) *kdr* mutations. The mutation was observed at all 13 sites and identified in *An. coluzzii*, *An. gambiae s.s*., and *An. arabiensis*. The overall allelic frequency was 71 % in both *An. coluzzii* and *An. gambiaes.s.*, and 40 % in *An. arabiensis*, indicating higher 1014F *kdr* frequency in *An. gambiaes.s.* and *An. coluzzii*, as compared to *An. arabiensis* (Table 5); it was not statistically significant when adjusted for sites (P = 0.23).

**Table 5:** *kdr* genotypes and frequency of *kdr* mutation in the *Anopheles coluzzii*, *An. gambiae s.s.* and *An. arabiensis*

	*Anopheles coluzzii*				*An. gambiae s.s.*				*An. arabiensis*			
	*Kdr* genotypes			F (*kdr*)	*Kdr* genotypes			*F* (*kdr*)	*Kdr* genotypes			*F* (*kdr*)
	RR	RS	SS		RR	RS	SS		RR	RS	SS	
Koulikoro	46	18	14	0.71	3	3	0	0.75	1	0	0	1.00
Baraoueli	9	5	2	0.72	34	9	15	0.66	10	1	3	0.75
Bla	22	12	14	0.58	8	3	5	0.59	13	7	11	0.53
Niono	16	16	8	0.60	17	8	5	0.70	7	6	5	0.56
Kita	2	7	10	0.29	5	4	12	0.33	0	3	43	0.03
Selingue	59	22	2	0.84	3	1	0	0.88				
Bougouni	22	4	3	0.83	47	10	8	0.80				
Djenne	50	28	13	0.70					1	4	0	0.60
Kadiolo	28	8	3	0.82	38	9	4	0.83	0	1	0	0.50
Bandiagara	58	19	6	0.81	0	1	0	0.50	0	1	0	0.50
Bankass	38	21	16	0.65	2	1	1	0.63	4	3	3	0.55
Bamako	33	37	16	0.60	2	2	1	0.60	2	0	0	1.00
Kati	63	21	12	0.77								
Total	446	218	119	0.71	159	51	51	0.71	38	26	65	0.40

Unadjusted Leu–Phe *kdr* frequency in *An. coluzzii* and *An. gambiae s.s.* was significantly higher than in *An. arabiensis* (P < 0.0001), but no significant difference was observed when the collection site was controlled for. No marked difference in the distribution and allelic frequency of 1014F *kdr* mutation was observed between the *An. coluzzii* and *An. gambiae s.s.* The frequency ranged from 29 to 84 % and 33 to 88 % in the *An. coluzzii* and *An. gambiae s.s*., respectively. The homozygous resistant genotype (RR) was dominant at 12 of the 13 sites (Table 5). Allelic frequencies in the *An. arabiensis* tested ranged from 3 to 100 %. There was no significant difference in the frequency of *Kdr*-w mutation between IRS and non-IRS areas (P = 0.63).

The presence of *ace*-*1*^*R*^ mutation was assessed at the four sites where bendiocarb and/or fenitrothion resistance were recorded, and the mutation was detected in all four sites at low allelic frequencies. The mean frequency was (8, 13 and 2 %) in *An. coluzzii*, *An. gambiae s.s*. and *An. arabiensis*, respectively (Table 6). It was significantly higher in *An. gambiae s.s*. when compared to *An. arabiensis* (P < 0.05), but did not significantly differ between *An. coluzzii* and *An. gambiae s.s.*, and between *An. coluzzii* and *An. arabiensis* (P > 0.05). Unlike *An. coluzzii* and *An. gambiae s.s*., where both homozygous and heterozygous *ace*-*1*^*R*^ mutation individuals were noted, only heterozygous mutation individuals were found in *An. arabiensis* (Table 6). No statistically significant difference was observed in the overall *ace*-*1*^*R*^ mutation allelic frequency and distribution of the homozygous resistant genotype between IRS and non- IRS areas in the population of *An. gambiaes.l.* in the study areas (P > 0.05).

**Table 6:** *ace*-*1* ^*R*^ genotypes and frequency of *ace*-*1* ^*R*^ mutation in the *Anopheles coluzzii*, *An. gambiae s.s.* and *An. arabiensis*

Study site	*An. coluzzii*				*An. gambiae s.s.*				*An. arabiensis*			
	*ace*-*1* ^*R*^ genotypes			F (*ace*-*1* ^*R*^)	*ace*-*1* ^*R*^ genotypes			*F* (*ace*-*1* ^*R*^)	*ace*-*1* ^*R*^ genotypes			*F* (*ace*-*1* ^*R*^)
	RR	RS	SS		RR	RS	SS		RR	RS	SS	
Bla	1	6	44	0.08	0	3	14	0.09	0	3	29	0.05
Kita	0	4	18	0.09	1	3	18	0.11	0	1	55	0.01
Bougouni	0	2	27	0.03	1	21	43	0.18				
Kadiolo	1	6	34	0.10	1	10	47	0.10	0	0	1	0.00
Total	2	18	123	0.08	3	37	122	0.13	0	4	85	0.02

### Vector taxonomy by site

When *An. gambiae s.l*. from all sites (n = 1287) were identified to species using PCR (Table 7) *Anopheles coluzzii*, *An. gambiae s.s*. and *An. arabiensis* were the only three members of the *An. gambiae* complex identified. Overall, there was a predominance of *An. coluzzii* (66.43 %) followed by *An. gambiae s.s.* (21.91 %) and *An. arabiensis* (11.66 %) in the study areas. *Anopheles coluzzii* predominated at nine of the 13 sites and *An. gambiae s.s.* predominated at three of the 13 sites. However, in Kita, *An. arabiensis* was found at slightly higher frequencies (56 %) than *An. coluzzii* (22 %) and *An. gambiae s.s*. (22 %) (Table 7). *Anopheles arabiensis* was not detected in three of the 13 sites (Kati, Selingue, and Bougouni). Similarly, *An. gambiae s.s*. was not detected in two out of 13 sites (Kati and Djenne).

**Table 7:** *Anopheles gambiae s.l.* species identification by site

Sites	No Tested	*Anopheles coluzzii*	*An. gambiae s.s.*	*An. arabiensis*
Koulikoro	96	88	7	1
Kati	100	100	0	0
Baraoueli	100	21	60	19
Bla	100	51	17	32
Niono	100	42	37	21
Kita	100	22	22	56
Selingue	100	95	5	0
Bougouni	94	29	65	0
Djenne	100	94	0	6
Kadiolo	100	41	58	1
Bandiagara	100	98	1	1
Bankass	97	82	5	10
Bamako	100	92	5	3
Total	1287	855	282	150

## Discussion

To ensure the success of malaria vector control efforts and malaria elimination in Africa, it is critical that a strategic plan, informed by comprehensive monitoring and evaluation of resistance, be in place [27, 28]. The President’s Malaria Initiative (PMI) has supported this approach in Mali, focusing on areas where insecticide-based vector control measures (IRS and LLINs) have been deployed. One advantage of this ‘dual’ approach is that in addition to reducing transmission, and hence malaria burden, IRS, with its ability to draw on multiple classes of insecticide, can be used to manage the emergence of insecticide resistance, especially PY resistance that threatens the efficacy of LLINs [2, 3, 29].

There are two main reasons for ongoing support of vector insecticide resistance. First, information on malaria vector insecticide-resistance status is a key input to the decision process surrounding the choice of IRS insecticide. Therefore, PMI has supported vector insecticide-resistance surveillance to inform this issue, specifically the relative frequency of phenotypic resistance, by insecticide class. A second important programme issue informed by these data is the distribution and intensity of vector-pyrethroid resistance and its relationship to LLIN impact. There has been a universal coverage target for LLIN distribution since 2011. The spread and intensification of pyrethroid resistance threatens this strategy. Given the growing threat of insecticide resistance it is essential that up-to-date data on the magnitude and distribution of insecticide resistance be collected. Currently in Mali, PMI supports resistance monitoring annually in IRS target areas to inform the selection of an effective class of IRS insecticide. This study was conducted to expand resistance monitoring to 13 sites across the central, south, and southwestern parts of the country.

The utility of routine monitoring to update vector-insecticide resistance status can be seen by comparing recent (2009) data from WHO-AFRO-Mali [30] to these study results. Prior to our investigation, resistance to DDT, deltamethrin, and lambda-cyalothrin was reported from four, three, and seven out of eight sites, respectively, that were part of this study. However, for fenitrothion (OP) and bendiocarb (CA), all the vector populations tested were shown to be susceptible in 2009. The present results update this picture by showing that except in one site, Bougouni, where there was a possibility of resistance to deltamethrin, *An. gambiae s.l.* populations from all other tested sites were resistant to DDT, lambda-cyhalothrin, and deltamethrin. These results suggest cross-resistance between DDT and PY class insecticides exists, probably due to the *kdr* mutation. Evidence of fenitrothion resistance was seen in only one out of 13 sites in this study. While there was evidence of bendiocarb resistance at four of 13 sites, this is the first time that bendiocarb (CM) and fenitrothion (OP) resistance has been reported in Mali.

Insecticide selection pressure exerted on vector populations may explain the rapid spread of resistance. DDT is no longer officially sanctioned in Mali, neither for use in public health nor agriculture. Permethrin and deltamethrin, however, are used for malaria control through Ministry of Health (MOH)/NMCP distribution of LLINs, and lambda-cyhalothrin and deltamethrin were used for IRS in Koulikoro and Bla, two of the 13 sites studied from 2008 to 2009 and in 2010, respectively. From 2011 to 2013 bendiocarb was used for IRS in 3/13 districts, with the addition of Baraoueli to the IRS programme in 2011. In 2014, Bla and Baroueli were sprayed with pirimiphos-methyl, while Koulikoro was sprayed with bendiocarb.

All public health classes of insecticides are also used in agriculture, especially in cotton growing areas in Kita, Bougouni and Kadiolo, three of 13 sites described in this report. In this regard, it is interesting to note that there was *An. gambiae s.l.* resistance to bendiocarb (CA) observed in three places, and to fenitrothion (OP) in Kadiolo, where those insecticides were used for agriculture but not for public health activities. The intensive use of insecticide to control agriculture pests [31] may contaminate mosquitoes breeding sites, thus exerting significant and constant selection pressure on *Anopheles* larval populations. Such an effect might explain the emergence of insecticide resistance in malaria vector populations before they ‘see’ insecticide-based vector control interventions with any class of insecticides. The only way to detect this ‘stealth’ appearance of resistance is through monitoring. The emergence of resistance in populations of *An*. *gambiae* to common classes of insecticides used in public health has been reported in many countries in Africa, including Côte d’Ivoire [10, 32], Kenya [33], Benin [32, 34, 35], Niger [4], Burkina Faso [9], Mali [16], Nigeria [36, 37], South Africa [38] and Cameroon [39].

In addition to documenting phenotypic resistance, this study provides information on the frequency and distribution of common physiological resistance mechanisms such as the *kdr*-*w* mutation, probably one of the most important mechanisms for pyrethroid and DDT resistance. The significance of this finding is the identification of the *kdr*-*w* allele in *An. arabiensis* in Mali, in addition to *An. coluzzii*, *An. gambiaes.s*., previous reported from Mali. This finding is in agreement with previous reports from several other African countries that indicated the widespread of *kdr*-*w* mutations in the three major vector species of the *An*. *gambiae* complex. In a recent study conducted in Mali, Norris et al. [40] elucidated the dynamics of how *An. coluzzii* inherited the insecticide-resistance allele from the *An. gambiaes.s*., in areas of increased insecticide exposure due to high coverage of LLINs and the resistance genes subsequently spread in the population. In *An. arabiensiskdr*-*w* mutation is reported to have occurred through a de novo mutation event [41].

Increased selection pressure due to the increased (PY) LLIN coverage over time [42], the culture of using PY insecticides for crop protection in agriculture, IRS using PY class insecticides in three districts and even widespread use of pyrethrin-based aerosols, in combination, or alone might have been sufficient to drive *kdr*-*w* mutations to the high frequencies in *An. gambiaes.l.* Previous study results by Czeher et al. [4] indicated that large-scale countrywide distribution of LLINs led to an increased frequency of *kdr*-*w* mutations in Niger. Use of PYs at the household level and in small vegetable cultivation has also been reported to drive the *kdr* mutation to a higher frequency in Mali [16].

The *ace*-*1*^*R*^ allele that confers resistance to OPs and CAs [43] was present in four localities (Bla, Kita, Bougouni, and Kadiolo) at lower frequencies than *kdr*-*w*. Some mosquitoes were found carrying both resistant alleles simultaneously. OPs nor CAs have been deployed to Kita, Bougouni, and Kadiolo for malaria vector control but OPs are commonly used for crop protection. Indoor residual spraying with CAs was implemented in 2011 and 2012 in Bla before this study was conducted. This might explain the *ace*-*1*^*R*^ mutation observed in those sites. The frequency and distribution of the *ace*-*1*^*R*^ allele in the other study sites are unknown and further investigation is required to map the distribution and gain information on the frequency of the allele from nationally representative sites and further understand its linkage with use of pesticides for agriculture.

Although the data did not allow us to assess whether there was any association between *kdr* and *ace*-*1*^*R*^ mutations and phenotypic resistance, previous studies have established association between target site mutation and phenotypic resistance [5, 10, 44]. Apart from target site resistance, data on levels of metabolic resistance mechanisms suggest that these might have contributed to the overall profile of insecticide resistance observed in Mali. Elevated levels of GST activity were detected in eight of nine sites. GSTs breakdown DDT and catalyze PY induced lipid peroxidation [45, 46]. The widespread DDT and PY resistance observed in Mali might, therefore, be due to the complementary effect of overexpressed GST and high frequency *kdr*–*w* mutations.

An overall increase in cytochrome P450 monooxygenases and elevated levels of non-specific esterases (NSE) activity were also detected in two and four out of 9 sites, respectively. Elevated NSE activity has been found to play an important role in OP and CA resistance in a number of arthropod species, including mosquitoes [46]. Similarly, overexpressed cytochrome P450 monooxygenases has been reported to have an association with insect resistance to DDT and PYs [46]. Hence, these two enzymes, where overexpressed, might have contributed to the insecticide resistance frequency observed in Mali.

## Conclusion

The results of this study revealed wide distribution of PY and DDT resistance in the population of *An. gambiae s.l.* in Mali. Resistance to CAs and OPs was also detected in some study sites. The study also demonstrated that multiple insecticide-resistance mechanisms have evolved in *An. coluzzii*, *An. gambiaes.s*. and *An. arabiensis* in Mali. The extent and variety of phenotypic resistance and the physiological mechanisms associated with it, serve as a ‘wake-up call’ for ongoing support of evidenced-based decision making around insecticide-based malaria control efforts. The results of this study highlight the importance of routine resistance monitoring to update the information base for rational deployment of the existing tools for effective control of malaria in Mali. The implications and operational impact of resistance to malaria control efforts needs to be urgently evaluated. Appropriate control strategies need to be put in place in a context of Insecticide Resistance Management. Innovative vector control tools that include new active ingredients for IRS and LLINs might be needed to complement or replace the existing strategies in areas of vector resistance.

## Abbreviations

AIRS: Africa indoor residual spraying
Ace-1R: acetylcholinesterase resistance
AChE: acetycholinesterase
CAs: carbamates
DDT: dichlorodiphenyltrichloroethane
GSTs: glutathione S-Transferases
IRS: indoor Residual Spraying
Kdr: knock-down resistance
Leu–Phe: leucine–phenylalanine
LLINs: long-lasting insecticide nets
OCs: organochlorines
Ops: organophosphates
PYs: pyrethroids
PCR: polymerase chain reaction
PMI: president malaria initiative
WHO: World Health Organization

## References

[1] The President’s Malaria Initiative Eighth Annual Report to Congress, President’s Malaria Initiative, Washington, DC, USA; 2014. http://www.pmi.gov/docs/default-source/default-document-library/pmi-reports/pmireport_final.pdf?sfvrsn=16. Accessed 12 Feb 2015.

[2] WHO: Global plan for insecticide resistance management in malaria vectors. World Health Organization Global Malaria Programme 2012; 13.

[3] Pluess B, Tanser FC, Lengeler C, Sharp BL. Indoor residual spraying for preventing malaria. Cochrane Database of Syst Rev. 2010; CD006657. 10.1002/14651858.CD006657.pub2 PMC6532743 20393950

[4] Czeher C, Labbo R, Arzika I, Duchemin J (2008). Evidence of increasing Leu–Phe knockdown resistance mutation in *Anopheles gambiae* from Niger following a nationwide long-lasting insecticide-treated nets implementation. Malar J..

[5] Ndiath MO, Sougoufara S, Gaye A, Mazenot C, Konate L, Faye O (2012). Resistance to DDT and Pyrethroids and increased *kdr* mutation frequency in *An. gambiae* after the implementation of permethrin-treated nets in Senegal. PLoS One.

[6] Ranson H, N’Guessan R, Lines J, Moiroux N, Nkuni Z, Corbel V (2011). Pyrethroid resistance in African anopheline mosquitoes: what are the implications for malaria control?. Trends Parasitol.

[7] Santolamazza F, Calzetta M, Etang J, Barrese E, Dia I, Caccone A (2008). Distribution of knockdown resistance mutations in *Anopheles gambiae* molecular forms in west and west-central Africa. Malar J..

[8] Ranson H, Abdallah H, Badolo A, Guelbeogo WM, Kerah-Hinzoumbe C, Yangalbe-Kalnone E (2009). Insecticide resistance in *Anopheles gambiae*: data from the first year of a multi-country study highlight the extent of the problem. Malar J..

[9] Diabate A, Baldet T, Chandare F, Akogbeto M, Guiguemde TR, Darriet F (2002). The role of agricultural use of insecticides in resistance to pyrethroids in *Anopheles gambiae s.l.* in Burkina Faso. Am J Trop Med Hyg.

[10] Ahoua Alou LP, Koffi AA, Adja MA, Tia E, Kouassi PK, Kone M (2010). The distribution of *ace*-*1*^*R*^ and resistance to carbamates and organophosphates in *Anopheles gambiae s.s.* populations from Cote d’Ivoire. Malar J..

[11] Yewhalaw D, Wassie F, Steurbaut W, Spanoghe P, Van Bortel W, Denis L (2011). Multiple insecticide resistance: an impediment to insecticide-based malaria vector control program. PLoS One.

[12] Maharaj R, Mthembu DJ, Sharp BL (2005). Impact of DDT re-introduction on malaria transmission in KwaZulu-Natal. S Afr Med J.

[13] N’Guessan R, Corbel V, Akogbeto M, Rowland M (2007). Reduced efficacy of insecticide-treated nets and indoor residual spraying for malaria control in pyrethroid resistance area, Benin. Emerg infect Dis..

[14] Strode C, Donegan S, Garner P, Enayati AA, Hemingway J (2014). The impact of pyrethroid resistance on the efficacy of insecticide-treated bed nets against African Anopheline mosquitoes: systematic review and meta-analysis. PLoS Med..

[15] Sogoba N, Vounatsou P, Bagayoko MM, Doumbia S, Dolo G, Gosoniu L (2007). The spatial distribution of *Anopheles gambiae* sensu stricto and *An. arabiensis* (Diptera: Culicidae) in Mali. Geospat Health..

[16] Fanello C, Petrarca V, della Torre A, Santolamazza F, Dolo G, Coulibaly M (2003). The pyrethroid knockdown resistance gene in the *Anopheles gambiae* complex in Mali and further indication of incipient speciation within *An. gambiae s.s*. Insect Mol Biol..

[17] Tripet F, Wright J, Cornel A, Fofana A, Mcabee R, Meneses C (2007). Longitudinal survey of knockdown resistance to pyrethroid (*kdr*) in Mali, West Africa, and evidence of its emergence in the Bamako form of *An. gambiae**s.s*. Am J Trop Med hyg..

[18] WHO (1998). Test procedures for insecticide resistance monitoring in malaria vectors, bio-efficacy and persistence of insecticides on treated surfaces.

[19] WHO (2013). Test procedures for insecticide resistance monitoring in malaria vectors mosquitoes.

[20] Diagne N, Fontenille D, Konate L, Faye O, Lamazana MT, Legros F (1994). Les anophèles du Sénégal. Liste commentée et illustrée. Bull Soc Pathol Exot.

[21] Hemingway J. Techniques to detect insecticide resistance mechanisms (field and laboratory manual). Document WHO/CDS/CPC/MAL/98.6. World Health Organization, Geneva, Switzerland. http://apps.who.int/iris/bitstream/10665/83780/1/WHO_CDS_CPC_MAL_98.6.pdf (1998). Accessed 8 Jan 2015

[22] Scott JA, Brogdon WG, Collins FH (1993). Identification of single specimens of the *Anopheles gambiae* complex by the polymerase chain reaction. Am J Trop Med Hyg.

[23] Favia G, Lanfrancotti A, Spanos L, Siden-Kiamos I, Louis C (2001). Molecular characterization of ribosomal DNA polymorphisms discriminating among chromosomal forms of *Anopheles gambiae**s.s*. Insect Mol Biol.

[24] Martinez-Torres D, Chandre F, Williamson MS, Darriet F, Berge JB, Devonshire AL (1998). Molecular characterization of pyrethroid knockdown resistance (kdr) in the major malaria vector *Anopheles gambiae**s.s*. Insect Mol Biol.

[25] Weill M, Malcolm C, Chandre F, Mogensen K, Berthomieu A, Marquine M (2004). The unique mutation in *ace*-*1R* giving high insecticide resistance is easily detectable in mosquito vectors. Insect Mol Biol.

[26] Coetzee M, Hunt RH, Wilkerson R, Della Torre A, Coulibaly MB, Besansky NJ (2013). *Anopheles coluzzii* and *Anopheles amharicus*, new members of the *Anopheles gambiae* complex. Zootaxa..

[27] WHO: *Global Malaria Action Plan World Health Organization*. Geneva, Switzerland: World Health Organization (2009).

[28] The malERA consultative group on vector control (2011). A research agenda for malaria eradication: vector control. PLoS Med.

[29] Lengeler C (2004). Insecticide-treated bed nets and curtains for preventing malaria. Cochrane Database Syst Rev.

[30] WHO AFRO-Mali. Rapport des activités sur le contrôle et la biologie des vecteurs. GATES/OMS PROJET. 2009.

[31] Dinham B (2003). Growing vegetables in developing countries for local urban populations and export markets: problems confronting small-scale producers. Pest Manag Sci.

[32] Elissa N, Mouchet J, Riviere F, Meunier JY, Yao K (1993). Resistance of *Anopheles gambiae* s.s. to pyrethroids in Côte-d’Ivoire. Ann Soc Belg Med Trop.

[33] Vullule JM, Beach RF, Atielic FK (1999). Mcallister Brogdon WG, Roberts JM. Elevated oxidase and esterase levels associated with permethrin tolerance in *Anopheles gambiae* from Kenyan villages using permethrin-impregnated nets. Med Vet Entomol.

[34] Corbel V, N’Guessan R, Brengues C, Chandre F, Djogbénou L, Martin T (2007). Multiple insecticide resistance mechanisms in *Anopheles gambiae* and *Culex quinquefasciatus* from Benin, West Africa. Acta Trop..

[35] Rock A, Agossa F, Ossè R, Oussou O, Aïzoun N, Oké-Agbo F (2013). Bendiocarb resistance in *Anopheles gambiae**s.l.* populations from Atacora department in Benin, West Africa: a threat for malaria vector control. Parasit Vectors.

[36] Awolola TS, Brooke BD, Koekemoer LL, Coetzee M (2002). Resistance of the malaria vector *Anopheles gambiae s.s. to* pyrethroid insecticides, in south-western Nigeria. Ann Trop Med Parasitol.

[37] Oduola AO, Idowu ET, Oyebola MK, Adeogun AO, Olojede JB, Otubanjo OA (2012). Evidence of carbamate resistance in urban populations of *Anopheles gambiae**s.s.* mosquitoes resistant to DDT and deltamethrin insecticides in Lagos, South-Western Nigeria. Parasit Vectors..

[38] Hargreaves K, Koerkemoer LL, Brooke B, Hunt RH, Mthembu J (2000). Coetzee. *Anopheles funestus* resistant to pyrethroid insecticides in South Africa. Med Vet Entomol.

[39] Etang J, Manga L, Chandre F, Guillet P, Fondjo E, Mimpfoundi R (2003). Insecticide susceptibility status of *Anopheles gambiae s.l.* (Diptera: Culicidae) in the Republic of Cameroon. J Med Entomol.

[40] Norris LC, Main BJ, Lee Y, Collier TC, Fofana A, Cornel AJ (2015). Adaptive Introgression in an African malaria mosquito coincident with the increased usage of insecticide-treated bed nets. Proc Natl Acad Sci USA.

[41] Diabate A, Brengues C, Baldet T, Dabire KR, Hougard JM, Akogbeto M (2004). The spread of the *Leu*-*Phe kdr* mutation through *Anopheles gambiae* complex in Burkina Faso: genetic introgression and de novo phenomena. Trop Med Int Health..

[42] Center for Studies and Statistics Information. Demographic health survey 2012–2013: Bamako, Mali. http://dhsprogram.com/pubs/pdf/FR286/FR286.pdf (2014). Accessed 5 Jan 2015.

[43] IRAC. Prevention and management of insecticide resistance in vectors of public health importance. The Insecticide Resistance Action Committee (IRAC). 2011.

[44] Kabula B, Kisinza W, Tungu P, Ndege C, Batengana B, Kollo D (2014). Co-occurrence and distribution of East (L1014S) and West (L1014F) African knock down resistance in *Anopheles gambiae* sensu lato population in Tanzania. Trop Med Int Health..

[45] Hemingway J, Hawkes NJ, McCarroll L, Ranson H (2004). The molecular basis of insecticide resistance in mosquitoes. Insect Biochem Mol Biol.

[46] Vontas JG, Small GJ, Hemingway J (2001). Glutathione S-transferases as antioxidant defense agents confer pyrethroid resistance in Nilaparvata lugens. Biochem J.

